# Healthy minds index, Spanish version of core dimensions of wellbeing

**DOI:** 10.1371/journal.pone.0328361

**Published:** 2025-08-14

**Authors:** Humberto Charles-Leija, Tammi R. A. Kral, Raquel Tatar, Richard J. Davidson, Carlos G. Castro, Rosalinda Ballesteros-Valdés, Masaya Okamoto

**Affiliations:** 1 Instituto del Propósito y Bienestar Integral, Universidad Tecmilenio, Mexico and Business School, Tecnologico de Monterrey, Mexico; 2 Healthy Minds Innovations, Madison, Wisconsin, United States of America; 3 Center for Healthy Minds, University of Wisconsin–Madison, Madison, Wisconsin, United States of America; 4 Department of Psychology, University of Wisconsin–Madison, Madison, Wisconsin, United States of America; 5 Instituto del Propósito y Bienestar Integral, Universidad Tecmilenio, México; School of Nursing Sao Joao de Deus, Evora University, PORTUGAL

## Abstract

Psychological wellbeing is a crucial aspect of human flourishing, yet validated assessment tools are needed across diverse linguistic and cultural contexts. This study aimed to adapt and validate the Healthy Minds Index (HMx) for Spanish-speaking populations (HMx-SV). The HMx-SV assesses four core dimensions of wellbeing: awareness, connection, insight, and purpose. We conducted exploratory and confirmatory factor analyses on data from two independent samples of university students (N = 362 and N = 1,594). The findings confirmed a four-factor structure, with strong internal consistency and convergent validity with established wellbeing measures. The HMx-SV demonstrated robust psychometric properties, making it a reliable and valid tool for assessing wellbeing in Spanish-speaking populations. By expanding access to a culturally adapted instrument, this study contributes to the broader understanding and promotion of psychological wellbeing in diverse communities.

## Introduction

The pursuit of wellbeing and human flourishing has long been a central focus in psychology and related fields [1,2]. Recent advances in neuroscience and psychological research have highlighted the potential for deliberately cultivating wellbeing through targeted training of specific mental skills [3,4]. The original Healthy Minds Index (HMx) was developed as a comprehensive measure to assess four key dimensions of wellbeing that can be developed through training: awareness, connection, insight, and purpose [5]. The four dimensions of the HMx represent distinct yet interrelated aspects of psychological functioning that contribute to overall wellbeing [4,6].

### Awareness

Awareness, the first dimension, involves the ability to focus on present-moment experiences, both internal and external, with an enhanced attentiveness to stimuli, thoughts, emotions, and bodily sensations [7]. This skill is fundamental to wellbeing as it allows individuals to engage more fully with their experiences and make conscious choices rather than operating on “autopilot” [8]. Research has shown that mindfulness-based interventions, which cultivate awareness, can lead to improvements in various aspects of mental health and wellbeing [9,10]. This improved focus can lead to better management of emotions and thoughts [4]. Previous studies have shown that body awareness positively influences subjective wellbeing [11]. It has been suggested that attention to internal sensations can have beneficial physiological and psychological consequences [11].

Attention to the present moment is a skill that significantly impacts individuals’ subjective wellbeing, and this skill can be taught through specifically designed techniques [12]. The practice of mindfulness meditation improves attentional control and awareness of internal experiences [13], which is crucial for emotional regulation and overall wellbeing. The ability to focus and remain attentive to the present moment, promoted by mindfulness meditation, positively impacts individuals’ quality of life and general wellbeing, especially in those with chronic conditions such as fibromyalgia [12].

### Connection

Connection, the second dimension, involves a sense of care and benevolence towards others, fostering supportive relationships, and positive social interactions [14]. The key skills for enhancing relationships with others include showing appreciation, kindness, and compassion. Positive social connections are consistently associated with greater wellbeing and better health outcomes [15,16]. The ability to form and maintain supportive relationships can be enhanced through training in social-emotional skills such as empathy and compassion [17,18]. By fostering a sense of connection, individuals can build resilience and access social support, both of which are crucial for maintaining wellbeing in the face of life’s challenges [19]. It has also been documented that compassion reduces stress and loneliness while increasing wellbeing [18].

Doing good for others often can help us feel better. Voluntary attitudes and actions intended to help, support, comfort, or care for others positively impact wellbeing [20]. Previous studies have noted that good personal relationships are as fundamental to subjective wellbeing as nutrition and temperature regulation [21]. It is important to note that the scale used in the present study measures the willingness to build good relationships with others and to practice kindness and compassion, rather than the quality of personal relationships.

Along the same lines, various studies have identified that feeling empathy for others, and even for oneself, contributes to wellbeing [22]. Other research has highlighted that empathy is a behavior that can be influenced through interventions, even brief ones [23]. In the context of medical students, it has been shown that empathy can be trained through artistic activities [23]. It is important to emphasize the motivation behind acts of kindness, such as kindness itself or prosocial purchases (spending on others rather than oneself); kindness practiced with a genuine desire to help others has a greater impact than when it is practiced benefiting oneself [24].

While various studies have demonstrated that acts of kindness and generosity positively impact one’s own wellbeing, others have suggested that simply wishing well for others can also enhance wellbeing, not just performing kind acts. This idea has been supported by studies focusing on a meditation practice known as “loving-kindness” meditation [25]. This type of meditation, which originates from Buddhist practice and has been practiced for over 2,500 years, aims to develop feelings of love and compassion toward oneself and others [26]. In loving-kindness meditation, the practitioner first directs kindness toward themselves, then toward loved ones, acquaintances, strangers, and ultimately toward all beings [26]. It has been documented that practicing compassionate kindness can increase empathy in healthcare professionals [26].

### Insight

Insight, the third dimension, refers to self-knowledge and the ability to understand one’s own psychological processes, including feelings and motivations. This self-inquiry skill allows individuals to recognize patterns in their thoughts, emotions, and behaviors, enabling more effective self-regulation [27,28]. Developing insight can lead to greater emotional intelligence and improved decision-making, both of which contribute to overall wellbeing [29,30]. Psychotherapeutic approaches that cultivate insight, such as cognitive-behavioral therapy (CBT), have been shown to be effective in treating various mental health conditions and improving quality of life [31,32]. Developing introspection requires training skills such as self-awareness. Introspection allows the mind to more easily receive new information and ideas. Insight is associated with greater self-understanding coupled with a positive evaluation of one’s own life, and it is related to internal awareness and acceptance of one’s own feelings and experiences [33].

Previous studies have identified that insight is strongly associated with various measures of wellbeing, both from the perspective of overall life evaluation and eudaimonic aspects, such as self-acceptance and a sense of purpose in life. Insight can be developed through meditation practices, cognitive therapy, or guided self-reflection processes such as journaling [34]. Mindfulness practices enable focused attention on oneself with an attitude of acceptance and non-judgment, which serves as the foundation for the development of insight.

### Purpose

Purpose, the final dimension, involves having a sense of meaning and direction in life [35]. A strong sense of purpose has been linked to numerous positive outcomes, including better physical health, increased resilience, and greater life satisfaction [36,37]. Interventions that help individuals identify and pursue meaningful goals have been shown to enhance wellbeing and reduce symptoms of depression [38,39]. By cultivating a sense of purpose, individuals can find motivation and fulfillment in their daily activities, contributing to a more satisfying and well-lived life [40,41]. It has also been shown that students who find greater meaning in their academic activities are more likely to complete their educational programs [42][NO_PRINTED_FORM]

Purpose is a future-oriented motivation that guides a person’s actions [43]. A lack of meaning in life has been linked to a higher risk of depression, anxiety, and other mental health issues. Conversely, having meaning in life is related to higher levels of job satisfaction, life satisfaction, and happiness [44]. Meaning in life is a crucial concept in wellbeing studies, as it marks one of the central differences between hedonic or short-term wellbeing (e.g., joy, entertainment) and psychological wellbeing and human flourishing [45]. Living in harmony with one’s values enhances a sense of self-integrity, which is critical for cultivating purpose and long-term motivation.

### Justification of measurement instruments

The validation of the Spanish version of the HMx (HMx-SV) is crucial for several reasons. First, it allows for cross-cultural comparisons of wellbeing dimensions, contributing to a more comprehensive understanding of psychological wellbeing across diverse populations [46,47]. Second, it provides a valuable tool for researchers and practitioners working with Spanish-speaking communities, enabling more accurate assessment and targeted interventions [48,49]. Moreover, future studies could explore whether wellbeing among English-speaking communities differs from that among Spanish-speaking communities (a topic that has been little explored in the literature) and minimize cultural and linguistic biases that may affect the validity of the results. This provides more complete data for formulating public policies that benefit the entire population. Finally, given the growing Hispanic population in many countries, including in the United States, having a validated Spanish version of the HMx can contribute to more inclusive and representative wellbeing research and practice [50]. The selection of instruments for validating the Spanish version of the HMx was based on their established reliability and validity in measuring the constructs of interest [5].

## Materials and methods

### Participants

All participants were affiliated with Tecmilenio University and were over 18 years old. Invitations were sent via email, specifying that the study aimed to validate an instrument measuring four aspects of wellbeing. By agreeing to participate, students also consented to provide sociodemographic information such as age, gender, and whether they spoke an indigenous language. All participants provided written consent. The study was approved by the Ethics Committee of Tecmilenio University under the name “Protocol number: 001-09/20/20. Study Product: Healthy Minds Index - Spanish Version.” Participants did not receive monetary compensation. They were given access to the Spanish version of an app called “Healthy Minds Program”, which is a digital training program. The first data collection took place in November 2020, after which some items were rephrased (the original items said, “I reflect on”, then it was modified to “I am clear about”). A second round of data collection occurred in February 2021. Data were collected using Qualtrics, the platform commonly used for surveying students at Tecmilenio University. The first sample size for the analysis in this study consisted of 362 participants. Since 2021, Tecmilenio students have used the Healthy Minds Program as part of a first-year university course. Students answer the HMx-SV questionnaire in the app. The first confirmatory analysis was carried out with data from 2021 (a survey carried out with the purpose of validating the instrument). A second confirmatory analysis was carried out with data available in the app. The second sample had 1,594 participants (app users). In both samples, all participants gave informed consent for their contribution.

### Instruments and measures

The original HMx instrument consists of 17 items: four related to awareness, six to connection, three to insight, and four to purpose. The 17 items used a five-point Likert scale. The awareness items ranged from “never” to “all the time,” the connection items from “not at all” to “completely” (for three items) and “never” to “always”, for three items, the insight items from “never” to “always,” and finally, the purpose items from “never” to “all the time”. The survey was administered in February of 2021 to form a first sample with over 362 students from Tecmilenio University aiming to explore the internal consistency and the construct validity through the structure of the scale. Construct validity was next assessed through the use of a second sample of 1594 used for confirmatory factor analyses.

The translation of the HMx followed the standard methodology for cross-cultural adaptation of self-report measures [51]. The translation process consisted of five stages: initial translation, synthesis of the translation, back-translation, expert validation, and pre-final version testing. Two bilingual experts participated in the back-translation of the scale, assessing conceptual and semantic equivalence until consensus was reached. Of the bilingual experts involved in translation, one possesses a PhD in psychology, while the other brings experience in cultural adaptation. The items with the least agreement were those related to the construct of insight, which required re-wording.

Table 1 presents a comparison between the original items of the Healthy Minds Index (HMx) and their Spanish translation (HMx-SV), along with the means and standard deviations of responses to the Spanish items from the first sample of participants.

**Table 1:** Items in Spanish and English.

Original Healthy Minds Index Item	Spanish Version Healthy Minds Index Item	Mean	Standard Deviation	Dependent Variables (Items)
When I want to focus, it’s easy for me.	Cuando quiero concentrarme, es fácil para mí.	4.28	1.18	a1
In general, I am able to focus when I’m reading.	En general, puedo concentrarme cuando leo.	4.17	1.26	a2
**I can notice my thoughts as soon as I have them.**	**Puedo notar mis pensamientos tan pronto como los tengo.**	**4.62**	**1.06**	**a3**
When some of my thoughts lead to other thoughts, I realize it while it is happening.	Cuando algunos de mis pensamientos me llevan a otros pensamientos, me doy cuenta mientras está pasando.	4.52	1.27	a4
I want all people to be happy, including people I don’t like.	Quiero que todas las personas sean felices, incluidas las que no me agradan.	4.99	1.13	c1
I care about the problems of people all over the world.	Me preocupan los problemas de las personas de todo el mundo.	4.29	1.25	c2
When I make decisions involving other people, I consider their best interests.	Cuando tomo decisiones que involucran a otras personas, considero sus mejores intereses.	4.78	1.03	c3
**I like all of the people I see from day to day.**	**Me gusta toda la gente que veo día a día.**	**4.40**	**1.25**	**c4**
**I actively take time to appreciate positive things about the people I see from day to day.**	**Me tomo tiempo activamente para apreciar las cosas positivas de las personas que veo día a día.**	**4.39**	**1.29**	**c5**
I believe that most people are doing the best they can.	Creo que la mayoría de la gente está haciendo lo mejor que puede.	4.47	1.24	c6
When I am interacting with someone, I reflect on how my feelings are causing me to treat them a certain way.	Cuando estoy interactuando con alguien, tengo claridad sobre cómo mis sentimientos hacen que lo trate de cierta manera.	4.62	1.18	i1
When I have a thought, I reflect on whether that thought is making me feel better or worse.	Cuando tengo un pensamiento, veo claramente si ese pensamiento me hace sentir mejor o peor.	4.54	1.27	i2
**I can change how I feel about a situation by changing my thoughts about that situation.**	**Tengo tanta claridad en mis emociones que puedo cambiar cómo me siento acerca de una situación cambiando mis pensamientos sobre esa situación.**	**4.34**	**1.22**	**i3**
I have general life goals that make my daily activities worth doing.	Tengo metas de vida generales que hacen que valga la pena hacer mis actividades diarias.	4.87	1.19	p1
I have a life purpose that guides my day-to-day choices.	Tengo un propósito de vida que guía mis elecciones diarias.	4.81	1.24	p2
I know what’s really important in my life.	Sé lo que es realmente importante en mi vida.	5.09	1.14	p3
I know what kind of life I want to lead.	Sé qué tipo de vida quiero llevar.	5.11	1.14	p4
The items removed during the adaptation process are highlighted in bold.				

### Convergence measures

For awareness, the Mindful Attention Awareness Scale (MAAS) was chosen due to its focus on measuring individuals’ concentration ability and awareness of their own thoughts [12]. The MAAS has been widely used in mindfulness research and has demonstrated good psychometric properties across various populations [8].

To assess connection, the Trust scale [52] was employed. Trust is a fundamental component of social connection and has been shown to be associated with various positive outcomes, including wellbeing and social capital [53]. The use of this instrument allows for a comparison of the HMx’s connection dimension with an established measure of interpersonal trust.

For insight, the Difficulties in Emotion Regulation Scale (DERS), is a solid scale, so it was selected [54,55]. The DERS assesses various aspects of emotion regulation, including awareness and understanding of emotions, which are closely related to the concept of insight in the HMx. Emotion regulation has been consistently linked to psychological wellbeing and mental health outcomes [28].

To evaluate the purpose dimension, the Meaning in Life Questionnaire [44] was utilized. The MLQ is a well-established measure that assesses both the presence of and search for meaning in life. It has demonstrated good psychometric properties and has been widely used in research on purpose and wellbeing.

Finally, to assess overall wellbeing and validate the HMx as a comprehensive measure, two established scales were employed: the Satisfaction with Life Scale [57] and the Human Flourishing Scale [58] named SWLS AND HFS, respectively. The SWLS is a widely used measure of global life satisfaction, while the HFS assesses various aspects of psychological wellbeing and flourishing. Both instruments have been extensively validated and used in wellbeing research across diverse populations [56,57].

### Procedure

We assessed construct validity, internal consistency, convergent validity, and divergent validity using SPSS version 21, STATA version 13, and the lavaan, semtools, and ggplot packages from the R programming,*version 4.1.2*. Convergent validity was established for each of the four dimensions of the Healthy Minds framework by calculating correlations for each domain with measures of similar or opposing constructs (in the case of insight). All relevant data are within the manuscript..

## Results

Table 2 shows the sociodemographic characteristics of a sample of 362 participants. Most of participants, 94.75%, are in the 18–24 age group, followed by 2.76% in the 25–40 age group, and 2.49% in the 41 years or older group. Regarding gender, 52.49% of the participants are female, 46.13% are male, and 1.38% preferred not to disclose their gender. Finally, in terms of Indigenous identity, 99.17% of participants do not identify as Indigenous, while 0.83% do. This distribution suggests that the sample is dominated by young adults, with a balanced gender representation and a majority not identifying as Indigenous.

**Table 2:** Descriptive demographic statistics (age, gender, indeigenous identity), n = 362.

		N	Percentage
Age	Group1: 18–24 years	343	94.75
	Group 2: 25–40 years	10	2.76
	Group3: 41 + years	9	2.49
Gender	Female	190	52.49
	Male	167	46.13
	Rather not to answer	5	1.38
Indigenous Identity	No	359	99.17
	Yes	3	0.83

### Reliability analysis

Once translated and adapted, data collection proceeded, the scale was administered to the participants, and their responses were gathered. With the data obtained, the reliability of the scale was evaluated by calculating Cronbach’s alpha coefficient, which measures the internal consistency of the scale. A Cronbach’s alpha value above 0.70 is considered acceptable, while values above 0.80 are considered very good [58]. The reliability analysis of our data yielded acceptable results. Below are the Cronbach’s Alpha values for the four main constructs. See Table 3.

**Table 3:** Reliability analysis (Cronbach’s alpha).

Factors	Alpha
Total Wellbeing	0.90
Awereness	0.74
Connection	0.78
Insight	0.71
Purpose	0.91

Table 3 presents the reliability analysis of the factors measured in the study, using Cronbach’s alpha as an indicator of internal consistency. The global Cronbach’s alpha for the four dimensions altogether was 0.88, which implies that the items within this factor were highly correlated with each other, but still within acceptable limits. The individual dimensions or factors also showed adequate levels of reliability, with awareness (α = 0.75), connection (α = 0.78), insight (α = 0.71), and purpose (α = 0.91). Overall, these results indicate that the factors had internal consistency ranging from adequate to excellent, providing a solid basis for interpreting the findings in terms of the dimensions measured. In general, these results suggest that the instrument used in the study was reliable, as the items were consistent with each other in measuring the proposed dimensions.

Table 4 shows the analysis of Cronbach’s alpha for the awareness factor in case of removing each of its individual items. It is observed that the removal of item hmi_a1 would result in a decrease of the alpha to 0.67, indicating that this item contributed significantly to the internal consistency of the factor. Similarly, the removal of items hmi_a2 and hmi_a3 would also decrease the alpha to 0.71 and 0.66 respectively, suggesting that both items are essential to maintain the reliability of the factor. However, the removal of item hmi_a4 would increase the alpha to 0.73, suggesting that this item did not contribute positively to the internal consistency of the factor and its exclusion could slightly improve its reliability.

**Table 4:** Alpha if the item (awareness) were removed.

*hmi_a1*	*0.67*
*hmi_a2*	*0.71*
*hmi_a3*	*0.66*
*hmi_a4*	*0.73*

Table 5 shows the Cronbach’s alpha analysis for the connection factor in the case of removing each of its individual items. The initial alpha of the factor was 0.78, and the removal of any item (hmi_c1 to hmi_c6) resulted in a slight decrease in alpha, with values ranging between 0.74 and 0.75. This indicates that each item contributed similarly to the internal consistency of the factor. In particular, items hmi_c4 and hmi_c5 showed that their removal would bring the alpha to 0.74, suggesting that these items were slightly less consistent compared to the others, but not significantly so. Overall, these results suggest that the connection factor had a fairly robust internal structure, and that all items contributed in a balanced way to its reliability, with no clear indication of problematic items that need revision or removal.

**Table 5:** Alpha if the item (connection) were removed.

hmi_c1	0.75
hmi_c2	0.75
hmi_c3	0.75
hmi_c4	0.74
hmi_c5	0.74
hmi_c6	0.75

Table 6 shows the Cronbach’s alpha analysis for the insight factor, which had an initial alpha of 0.71, if each of its individual items were removed. Removing item hmi_i1 would reduce the alpha to 0.62 and removing item hmi_i2 would further reduce it to 0.52, indicating that both items were essential to maintain the reliability of the factor. However, removing item hmi_i3 would not alter the alpha, which remained at 0.71. These results suggest that while hmi_i1 and hmi_i2 were crucial for the coherence of the insight factor, item hmi_i3 had a more modest impact if removed.

**Table 6:** Alpha if the item (insight) were removed.

hmi_i1	0.62
hmi_i2	0.52
hmi_i3	0.71

Table 7 shows the Cronbach’s alpha analysis for the purpose factor, which had an initial alpha of 0.90, if each of its individual items were removed. Removal of any item (hmi_p1 to hmi_p4) would result in a slight decrease in alpha to values between 0.87 and 0.88, indicating that each item contributed significantly to the high internal consistency of the factor. The removal of items hmi_p1 and hmi_p2 would reduce the alpha to 0.87, while the removal of hmi_p3 and hmi_p4 would decrease it to 0.88. These results suggest that all items were essential to maintain the reliability of the purpose factor, and although the removal of any item would result in a slight decrease in alpha, the internal consistency of the factor would still be high. Taken together, these results demonstrate that the purpose factor had a very robust internal structure, and each item contributed in a balanced and significant way to its reliability.

**Table 7:** Alpha if the item (purpose) were removed.

hmi_p1	0.87
hmi_p2	0.87
hmi_p3	0.88
hmi_p4	0.88

### Factor analysis

Multiple factor analyses were performed in order to explore and confirm the instrument’s structure. An exploratory factor analysis was conducted to explore the construct validity of the scale. This method helps determine if the number of items can be explained by a smaller set of underlying factors. The goal is to verify whether these factors correspond to the four theoretical constructs of the HMx-SV.

To ensure the appropriateness of factor analysis, the Kaiser-Meyer-Olkin (KMO) test was applied. A KMO value above 0.90 is considered excellent, above 0.80 is good, and above 0.70 is acceptable [59]. In this case, the KMO value was 0.91, indicating excellent sampling adequacy and confirming the suitability for factor analysis.

Bartlett’s test of sphericity was also performed to assess the applicability of a factor analysis model. A significant p-value (less than 0.05) suggests that factor analysis is appropriate [59]. For the dataset, the p-value of Bartlett’s test (Chi square) was < 0.01, confirming that the factor analysis model can be applied. See Table 8 for results of the exploratory factor analysis.

**Table 8:** KMO and Bartlett’s test results.

		First sample	Second sample
Kaiser-Meyer-Olkin		0.91	0.91
Bartlett	Chi-squared	3094.00	3094.00
	gl	136.00	136.00
	Sig.	<0.01	<0.01

**Fig 1:**
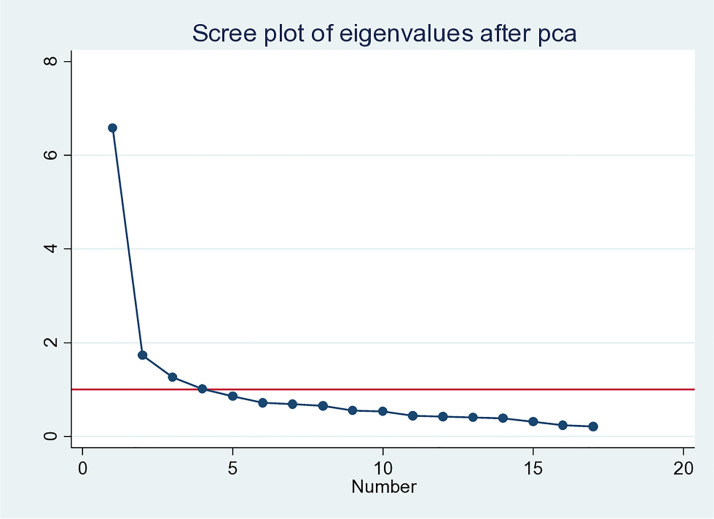
Eigenvalues and components (first sample left).

In exploratory factor analysis, it is generally recommended to preserve principal components with eigenvalues greater than 1. The principal components analysis (PCA) reveals that in the same fashion as the original English version of the scale, there are four key components that should be considered (see Fig 1). Altogether, the eigenvalues of these components account for 56.15% in first sample and 62.27% in second sample of the Spanish version of the total variance (see Table 9 to review all eigenvalues’ variance and cumulative percentages).

**Table 9:** Eigenvalues and total variance.

Components	Eigenvalues		
	Total	Variance %	Cumulative %
1	6.58	38.71%	38.71%
2	1.72	10.15%	48.86%
3	1.26	7.44%	56.30%
4	1.01	5.97%	62.27%
5	0.85		
6	0.71		
7	0.69		
8	0.65		
9	0.54		
10	0.53		
11	0.44		
12	0.42		
13	0.40		
14	0.39		
15	0.30		
16	0.24		
17	0.20		

Table 10 presents the factor loadings after varimax rotation of the items corresponding to each of the four main components. It includes only the items that were retained based on their factor loadings and the absence of significant cross-loadings (marked in bold). All factor loadings were above 0.40.

**Table 10:** Factor loadings after varimax rotation.

	1	2	3	4
hmi_a1			**0.59**	
hmi_a2			**0.53**	
hmi_a3			**0.58**	
hmi_a4			**0.46**	
hmi_c1				**0.51**
hmi_c2				**0.59**
hmi_c3				**0.54**
hmi_c4				**0.42**
hmi_c5	0.43	0.41		
hmi_c6		**0.45**		
hmi_i1		**0.54**		
hmi_i2		**0.62**		
hmi_i3		**0.44**		
hmi_p1	**0.81**			
hmi_p2	**0.84**			
hmi_p3	**0.79**			
hmsei_p4	**0.77**			

### Confirmatory analysis

A confirmatory factor analysis (CFA) was performed with a new sample of respondents.

Construct validity can be obtained through CFA techniques. For this motive, it is advisable to conduct Confirmatory Factor Analysis (CFA) with a second independent sample from the one used in Exploratory Factor Analysis (EFA). The main reason is that EFA is used to identify the underlying factor structure without imposing prior constraints, while CFA serves to validate that structure in a different sample, ensuring that the results are not specific to the first sample [60].

#### Outlier detection and handling.

The initial second sample consisted of 1,655 observations. To improve model fit and minimize undue influence on parameter estimates, outliers (extreme values in any direction) were identified and removed. A bivariate regression was conducted for the first pair of items, and residual scores were calculated and studentized (converted into Student’s r standard scores). Observations with *r* values exceeding ±2 were classified as outliers and excluded, resulting in a final sample of 1,594 observations.

#### Factor structure.

CFA indicates that the instrument meets the psychometric standards outlined in the literature. Research suggests that a model fits well when certain relative and absolute coefficients are achieved: a Comparative Fit Index (CFI) and Tucker-Lewis Index (TLI) greater than 0.90 are considered adequate. For the Root Mean Square Error of Approximation (RMSEA), values below 0.05 indicate excellent fit, below 0.06 a good fit, and below 0.08 an adequate fit. Additionally, a Standardized Root Mean Square Residual (SRMR) below 0.08 is recommended [61–63].

**Fig 2:**
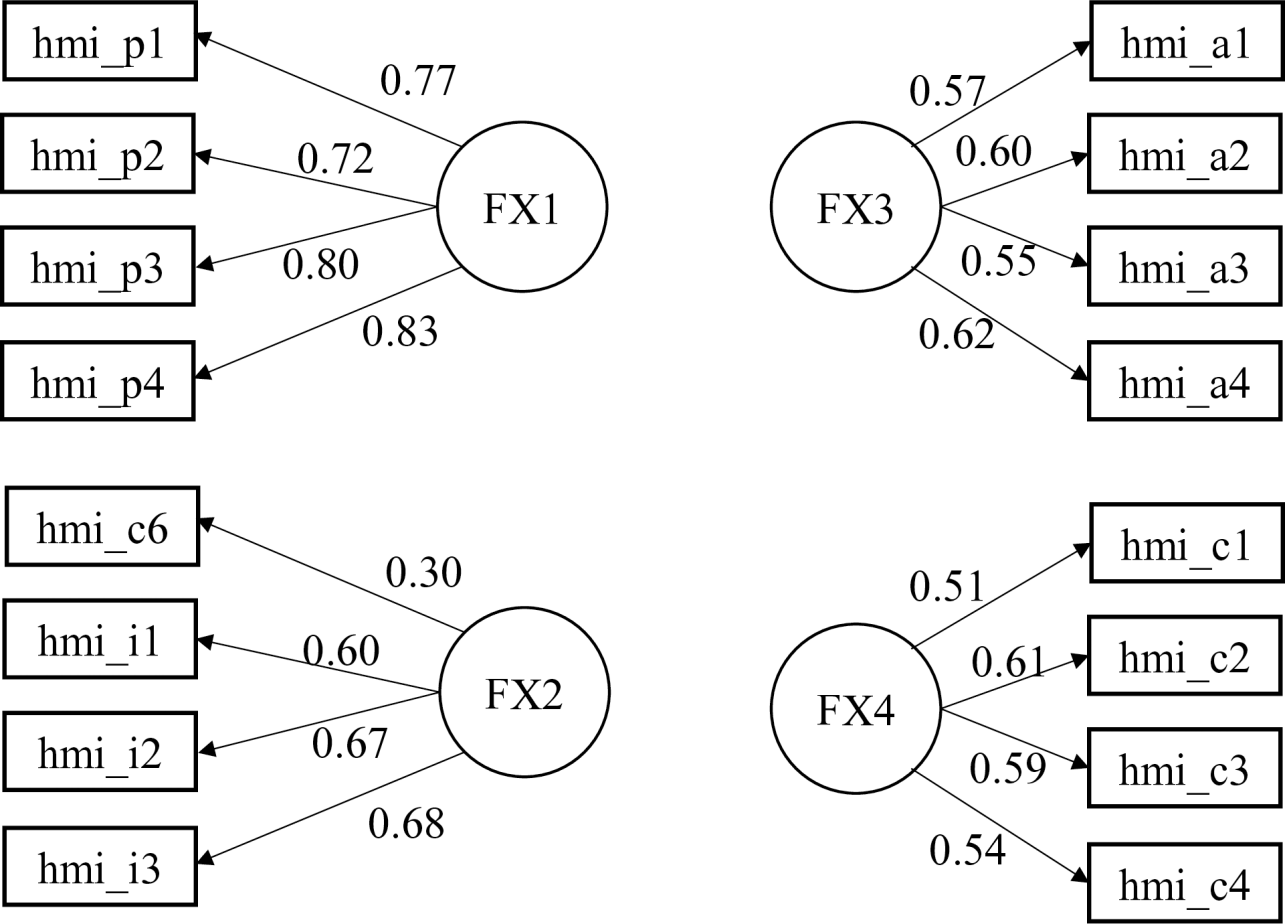
Graphic model of the HMx-SV structure confirmed by the CFA.

The current model, composed of four latent dimensions (constructs) and 16 observed variables, demonstrated an adequate fit, validating it as a reliable measurement tool. See Table 11 for the results of the CFA for each sample and Fig 2 to review the graphic model of the structure of the scale.

**Table 11:** Confirmatory factor analysis.

Factors	Items	N	TLI	CFI	RMSEA (p. value)	SRMR
4	16	Sample 2: 1594	0.932	0.947	0.053 (0. 12)	0.045

Table 12 shows the internal consistency of the items within the latent factors or dimensions before and after conducting the factor analyses. It was observed that consistency improved by reducing overall redundancy. Cronbach’s alphas changed slightly after relocating one item and removing another (i.e., *hmi_c6* was moved to the *Insight* dimension, and *hmi_c5* was removed from the analysis due to cross-loadings on two factors; see Table 10).

**Table 12:** Cronbach´s Alphas before and after factorial analyses.

Factor	Prior Alpha 4 factors & 17 items	New Alpha 4 factors & 16 items
Total Wellbeing	0.9	0.88
Awereness	0.74	0.74
Connection	0.78	0.71
Insight	0.71	0.74
Purpose	0.9	0.9
Overall redundancy diminished		

Fig 2 presents the graphic model of the scale’s structure as validated by the CFA. The items in all dimensions exhibit factor loadings ranging from acceptable to very good, except for item hmi_c6 in the Insight dimension. As noted earlier, this item was assigned to the Insight dimension based on its factor loading rather than to the Connection dimension, as in the original Anglo-Saxon version. This issue is discussed in greater detail in the discussion section.

Table 13 presents the correlations between the items associated with the four factors of interest from the HMI scale. These factors were compared against scales for measuring awareness (MAAS), connection (trust), insight (DERS), and purpose (MLQ), as well as two additional scales measuring subjective wellbeing (SWLS) and human flourishing (HF).

**Table 13:** Convergent validity with BEAT, CSRL, and UWES.

	hmi	hmi_a	hmi_c	hmi_i	hmi_p	maas(a)	trust (c)	ders (i)	mlq (p)	swls (sw)	flourishing (hf)
hmi		.77**	.85**	.79**	.79**	.16**	.39**	−.27**	.50**	.51**	.59**
hmi_a			.49**	.50**	.52**	.21**	.28**	−.25**	.34**	.36**	.40**
hmi_c				.62**	.50**	0.02	.40**	−0.09	.33**	.38**	.43**
hmi_i					.50**	.10*	.31**	−.21**	.41**	.39**	.45**
hmi_p						.21**	.25**	−.34**	.54**	.51**	.61**
maas(a)							−0.02	−.51**	0.07	.23**	.29**
trust (c)								−0.08	.30**	.38**	.35**
ders (i)									−.18**	−.43**	−.37**
mlq (p)										.46**	.58**
swls (sw)											.64**
flourishing (hf)											

It can be seen from Table 13 that some strong associations with other scales were evident (e;g. hmi-flourishing, while others were more modest (e.g., maas-hmi_c). The results indicate significant correlations and convergent validity for all factors with variables in their associated domain, and for the total Healthy Minds Index wellbeing score and measures of subjective wellbeing. Moreover, all associations for tests of convergent validity by domain were below the threshold of r = 0.60, reflecting discriminant validity and demonstrating that the constructs measured by the Healthy Minds Index are not redundant with comparison measures.

## Discussion

The primary objective of this study was to validate the Spanish version of the Healthy Minds Index (HMx-SV) in a population of university students in Mexico. The reliability and validity of the instrument were rigorously evaluated and demonstrated that the four theoretical factors of the HMx (awareness, connection, insight, and purpose) were maintained with slight modifications, showing both reliability (internal consistency) and validity. The constructs adapted to Spanish were psychometrically explored and confirmed.

The items initially showed very adequate levels of internal consistency. These reliability levels were consistent with those found in previous studies that have validated similar instruments in different contexts [51]. However, adjustments, such as the exclusion of one item, were necessary to achieve an adequate fit for the structure of the measurement model.

The decision to reassign item hmi_c6 from the “Connection” factor to the “Insight” dimension carries meaningful theoretical implications. The item, “I believe that most people are doing the best they can,” initially fits within a framework of prosocial attitudes, which would naturally align with Connection. However, its empirical loading onto the Insight factor in the Spanish-speaking context reveals a nuanced shift in interpretation: instead of primarily signaling interpersonal empathy, the item appears to reflect a cognitive reappraisal process—an internal belief system about others’ behaviors. This realignment suggests that in the cultural context of the Mexican university students surveyed, understanding and interpreting others’ actions is experienced more as an introspective judgment than a purely relational sentiment. It implies that culturally informed cognitive processes, such as the attribution of intent or effort, may be more integral to self-understanding (insight) than to social connection per se.

In contrast, the elimination of hmi_c5 (“I actively take time to appreciate positive things about the people I see from day to day”) due to cross-loadings across multiple factors—particularly Purpose and Insight—also holds conceptual weight. The item’s ambiguity in factor allegiance suggests that appreciation, as expressed here, may not uniquely capture the intended construct of Connection. Instead, participants might interpret such appreciation as reflecting deeper personal values (Purpose) or introspective habits (Insight). Its removal enhances construct clarity, helping ensure that each factor in the HMx-SV cleanly measures a distinct domain of wellbeing. Together, these adjustments illustrate the importance of cultural validation not just at the linguistic level but in the conceptual mapping of psychological constructs, highlighting how constructs like compassion or appreciation may function differently across cultural-linguistic populations.

Internal consistency was reassessed after testing construct validity, showing improved values due to reduced overall redundancy. It is important to note that Cronbach’s alpha depends on the construct being measured and varies based on item characteristics [64]. An important aspect to highlight is the contribution of the HMx-SV in measuring constructs that are less explored in the literature, such as insight. Including insight in the scale is valuable because it adds a unique and relevant dimension that has not been traditionally considered and has important implications for understanding and improving human well-being.

### Construct validity

As explained in the results section, the EFA indicated that item *hmi_c6* aligned more strongly with the insight dimension (factor loading = 0.45; see Table 10 in the results section). However, in the CFA, its loading was relatively low (factor loading = 0.30; see Fig 2 in the results section). When the SEM-CFA model was tested without this item, the overall fit deteriorated, leading to the decision to retain it. In this case, the phrasing of “I believe that most people are doing the best they can” appears more closely aligned with the insight dimension than with the connection dimension in the Mexican Spanish-speaking cultural context of the sample.

Moreover, the final set of sixteen items performed the best across the confirmatory models (SEM-CFA) conducted. These items selected for demonstrating strong factor loadings without cross-loadings in the EFA, showed good absolute and relative coefficients in the final SEM-CFA, confirming their validity. The confirmatory factor analysis (SEM-CFA) supported the theoretical four-factor structure of the Healthy Minds Index, with fit indices within the ranges considered appropriate and very good according to psychometric literature [62,63]. These results suggest that the theoretical model underlying the Healthy Minds Index is suitable for the studied population, confirming the construct validity of the instrument; It is worth noting that as a methodological precaution, this analysis was performed, in a different sample from the initial used for the EFA.

Convergent and divergent validity were assessed through correlations with established measures of similar constructs (MAAS, Trust, DERS, MLQ) and subjective wellbeing scales (SWLS, Flourishing). The results showed significant correlations in the expected directions, both within each domain and together for the total score of wellbeing across domains, which strengthens the convergent validity of the Healthy Minds Index.

In conclusion, the four core dimensions of the Healthy Mind Index—awareness, connection, insight, and purpose—can be measured with validity and reliability, and can also provide a valid measure of overall wellbeing when combined. The chosen instruments for measuring these dimensions were robust, reliable, and valid, ensuring that the Healthy Minds Index Spanish version can effectively capture the essential aspects of psychological health within these skills-based domains.

Most psychology studies rely on samples from WEIRD (Western, Educated, Industrialized, Rich, and Democratic) populations, which limits the generalizability of the results to all of humanity [65]. Validating psychological assessment tools in specific cultural contexts is essential to avoid misdiagnosis and ensure appropriate treatment strategies [66]. A study compared 33 countries and found that “tight” cultures (e.g., Japan) versus “loose” cultures (e.g., USA) show radical differences in variables like self-control and cooperation. This challenges some previous findings drawn solely from WEIRD samples and highlights the relevance of integrating other cultures into analyses in psychology and social sciences [67]. By validating the Healthy Minds Index in Spanish, this study contributes to a broader understanding of wellbeing and its determinants across different linguistic and cultural contexts.

### Limitations

One limitation of this study was the absence of invariance testing, which would determine whether this measurement model or scale functions equivalently across different populations or conditions (i.e., different educational levels). As a result, the study’s findings may be limited in terms of cross-group validity, measurement fairness, and generalizability, potentially introducing some bias. Future research is recommended to address these concerns. Despite this limitation, the instrument has been thoroughly validated using a range of other statistical modeling tools to test its reliability and validity as a psychometric measurement tool.

Another important aspect regarding the alpha values of the purpose domain is that some might argue that they are high enough to suggest redundancy. However, SEM-CFA modeling of purpose without the items that lowered Cronbach’s alpha values the most (*hmi_p1* or *hmi_p2*; see Table 7 in the results section) resulted in a deterioration of the overall fit—similar to what occurred when removing item *hmi_c6* due to its low factor loading in the SEM-CFA. Consequently, the decision was made to retain *hmi_p1* and *hmi_p2*, just as *hmi_c6* was retained for the same reason. These choices were deemed the most parsimonious and ultimately the most appropriate.

## Conclusions

The study confirms that the Spanish version of the Healthy Minds Index is a reliable and valid tool for measuring the four pillars of psychological wellbeing (awareness, connection, insight, and purpose) among university students in Mexico. The initial high internal consistency reflects an instrument with stable dimensions, but it is truly the results of the factor analyses that provide a solid foundation for the measure’s future use in research and practical applications within educational and clinical settings.

Validating the Healthy Minds Index in Spanish was a significant step toward understanding and promoting psychological wellbeing in Spanish-speaking contexts. The findings suggest that the four pillars were well-represented and consistently assessed by the instrument, which now has the advantage of being useful as a measurement scale for interventions based on these pillars.

Furthermore, the Spanish version of the Healthy Minds Index can serve as a valuable tool for future psychological wellbeing training programs, as it could prove useful for evaluating and monitoring interventions aimed at enhancing mindful awareness, interpersonal connections, introspective clarity, and a sense of purpose in individuals’ lives. The virtue of the Spanish version of the Healthy Minds Index to significantly correlate with established measures of wellbeing and related constructs suggests its potentiality to be integrated into standard assessment practices in positive psychology and wellbeing.

In conclusion, the validation of the Healthy Minds Index-Spanish-Version opens new avenues for research and practice in psychological wellbeing within Spanish-speaking communities. Educators, clinicians, and mental health professionals can use this instrument to develop and assess programs that foster holistic wellbeing, supporting individuals in building more meaningful and fulfilling lives. The study also emphasizes the importance of cultural adaptation and validation of measurement tools, ensuring that they accurately capture psychological constructs across diverse cultural contexts.

## Aknowledgements

We are grateful for the support provided throughout the validation process of this document to Bruno Zepeda, Carlos Mora and Mario Toledo.

## Supporting information

S1 Data Data and scripts. (ZIP)
